# Utilizing Two Populations Derived from Tropical Maize for Genome-Wide Association Analysis of Banded Leaf and Sheath Blight Resistance

**DOI:** 10.3390/plants13030456

**Published:** 2024-02-04

**Authors:** Shaoxiong Li, Fuyan Jiang, Yaqi Bi, Xingfu Yin, Linzhuo Li, Xingjie Zhang, Jinfeng Li, Meichen Liu, Ranjan K. Shaw, Xingming Fan

**Affiliations:** 1 College of Agriculture, Yunnan University, Kunming 650500, China; 15987701739@163.com (S.L.); lilinzhuo0606@163.com (L.L.); xingjiezhang2022@163.com (X.Z.); jinfengli1020@163.com (J.L.); shirleyliu1028@163.com (M.L.); 2 Institute of Food Crops, Yunnan Academy of Agricultural Sciences, Kunming 650205, China; jiangfuyansxx@126.com (F.J.); biyq122627@163.com (Y.B.); xingfuyin626@163.com (X.Y.); ranjanshaw@gmail.com (R.K.S.)

**Keywords:** maize, BLSB, *Rhizoctonia solani*, GWAS, candidate gene

## Abstract

Banded leaf and sheath blight (BLSB) in maize is a soil-borne fungal disease caused by *Rhizoctonia solani Kühn*, resulting in significant yield losses. Investigating the genes responsible for regulating resistance to BLSB is crucial for yield enhancement. In this study, a multiparent maize population was developed, comprising two recombinant inbred line (RIL) populations totaling 442 F8RILs. The populations were generated by crossing two tropical inbred lines, CML444 and NK40-1, known for their BLSB resistance, as female parents, with the high-yielding but BLSB-susceptible inbred line Ye107 serving as the common male parent. Subsequently, we utilized 562,212 high-quality single nucleotide polymorphisms (SNPs) generated through genotyping-by-sequencing (GBS) for a comprehensive genome-wide association study (GWAS) aimed at identifying genes responsible for BLSB resistance. The objectives of this study were to (1) identify SNPs associated with BLSB resistance through genome-wide association analyses, (2) explore candidate genes regulating BLSB resistance in maize, and (3) investigate pathways involved in BLSB resistance and discover key candidate genes through Gene Ontology (GO) analysis. The GWAS analysis revealed nineteen SNPs significantly associated with BLSB that were consistently identified across four environments in the GWAS, with phenotypic variation explained (PVE) ranging from 2.48% to 11.71%. Screening a 40 kb region upstream and downstream of the significant SNPs revealed several potential candidate genes. By integrating information from maize GDB and the NCBI, we identified five novel candidate genes, namely, *Zm00001d009723, Zm00001d009975, Zm00001d009566, Zm00001d009567*, located on chromosome 8, and *Zm00001d026376,* on chromosome 10, related to BLSB resistance. These candidate genes exhibit association with various aspects, including maize cell membrane proteins and cell immune proteins, as well as connections to cell metabolism, transport, transcriptional regulation, and structural proteins. These proteins and biochemical processes play crucial roles in maize defense against BLSB. When *Rhizoctonia solani* invades maize plants, it induces the expression of genes encoding specific proteins and regulates corresponding metabolic pathways to thwart the invasion of this fungus. The present study significantly contributes to our understanding of the genetic basis of BLSB resistance in maize, offering valuable insights into novel candidate genes that could be instrumental in future breeding efforts to develop maize varieties with enhanced BLSB resistance.

## 1. Introduction

Maize (*Zea mays* L.) stands as a primary cereal crop cultivated extensively worldwide, playing a dual role as a staple food source and a vital contributor to fodder and biofuel production. However, maize production faces persistent challenges from both biotic and abiotic stresses, resulting in substantial disruptions to yield and product quality [1]. Notably, banded leaf and sheath blight (BLSB), caused by the fungus *Rhizoctonia solani*, has emerged as a severe threat to maize crops. BLSB is a widespread disease that poses a significant threat to maize plants globally. This disease holds the potential to cause yield reductions ranging from 11% to 40% and, under severe conditions, can even lead to complete crop failure [2,3,4]. According to Chen [5], Songyang County in Zhejiang Province experiences a significant outbreak of BLSB every autumn. In 1983, the average incidence rate of BLSB was 41.2%, leading to a 9.3% loss in maize yield. By 1985, the incidence rate surged to 70.8%, causing a 16.5% yield loss. Tan et al. [6] documented a significant BLSB outbreak in the western mountains of Hubei Province in 1987. A survey conducted in Zigui County revealed that out of 185,000 SI of maize, 80,000 SI were affected, with 15,000 acres experiencing severe infection. The average incidence rate was 98.5%, with some plots recording a 100% incidence rate. The average disease index was 51.4%, peaking at 64.8%, resulting in an average yield loss of 18.8%. The primary causative agent, *Rhizoctonia solani* f. sp. *Sasakii Exner*, is prevalent in maize growing regions worldwide, with particular prominence in the southern parts of China and maize-growing areas in Southeast Asian countries [4]. BLSB, induced by *Rhizoctonia solani*, typically manifests in the leaf sheaths of the first to third nodes of maize plants. The lesions progress gradually upward, extending eventually to the corn ears and husks, where the characteristic sclerotia form at the lesion sites. *Rhizoctonia solani* can disrupt the vascular tissues of maize plants, impeding the normal transport of water and nutrients and resulting in a significant reduction in maize yield. This disease tends to thrive in warm and humid climatic conditions, especially when the temperature is maintained at 28 ± 2 °C and the relative humidity exceeds 88% [7,8].

*R. solani,* a soil-borne fungus with widespread geographical distribution and a broad host range, poses a significant challenge during maize cultivation. Currently, the primary method for controlling BLSB involves the use of chemical fungicides. However, this approach is becoming increasingly environmentally unsustainable and less acceptable to consumers due to heightened concerns about food safety and environmental protection. Consequently, the cultivation or development of maize varieties resistant to BLSB has become crucial. Despite this urgency, most existing maize varieties remain susceptible to *R. solani*, while only a few maize germplasms exhibit high resistance to BLSB. This limitation significantly hampers both the study of resistance mechanisms and the development of disease-resistant maize varieties [9]

Genome-wide association study (GWAS) is a highly effective mapping approach based on the principle of linkage disequilibrium (LD) for precisely mapping quantitative trait loci (QTL) that control complex traits. Initially proposed in 1996 [10], this approach was originally applied to analyze complex traits associated with human diseases. Through this method, researchers can precisely locate genomic regions linked to specific traits in crops, thereby advancing our understanding of the intricate mechanisms controlling these traits [11,12]. GWAS has emerged as a robust and efficient tool in genetic research, playing a pivotal role in deciphering the genetic basis of complex traits in crops [13,14] In recent years, scholars have increasingly employed GWAS to investigate loci controlling various traits in maize, including plant height (PH), ear length (EH) [10], yield [15], disease resistance [16], and grain dehydration [17]. Through GWAS, Li Ning [18] successfully identified a gene, *ZmFBL4*, associated with banded leaf and sheath blight. Numerous studies have successfully identified QTLs related to crucial agronomic traits, thereby showcasing the practicality and efficiency of GWAS [19,20,21,22]. In crops like maize, GWAS has proven invaluable in exploring key genes and identifying potential disease-resistant genes. The progression of these studies unequivocally underscores GWAS as a powerful tool for effectively establishing associations between markers and phenotypes.

In recent years, significant progress has been made in investigating genetic resources related to resistance against BLSB in maize. Researchers have identified maize varieties, such as Jinyu 506 [23], Zhongke Yu505 [24], and Endan801 [25], that exhibit enhanced resistance to BLSB. Wu et al. [26] reported that the novel sweet corn variety ‘Huawang Sweet No.7’ displays high resistance to leaf blight and moderate resistance to BLSB. However, currently, maize varieties identified with relative resistance to *R. solani* are predominantly moderately resistant, with no highly resistant or immune maize varieties having been discovered. Therefore, it is crucial to explore additional maize germplasm resources exhibiting higher resistance to BLSB and understand the genetic mechanisms underlying such resistance, and this holds significant importance for breeding maize varieties resistant to BLSB [27]. In this study, we utilized the tropical inbred lines CML444 and NK40-1 as resistant parents, crossing them with the excellent maize inbred line Ye107 from the Reid heterotic group to develop a multi-parent population comprising 442 F8 recombinant inbred lines (RILs). Subsequently, the multi-parent population underwent a GWAS analysis. The study’s objectives were to (1) identify SNPs associated with BLSB resistance through genome-wide association analysis, (2) explore candidate genes regulating BLSB resistance in maize, and (3) investigate pathways involved in BLSB resistance and discover key candidate genes through Gene Ontology (GO) analysis.

## 2. Results

### 2.1. Phenotypic Data Analysis

Screening of the two RIL populations, pop1 and pop2, against BLSB was conducted at two locations; Yanshan County, Yunnan Province, in the years 2021 and 2022, and Jinghong City, Yunnan Province, in the year 2022, with corresponding data collected. Descriptive statistics of the phenotypic data for the two RIL populations screened against BLSB are presented in Table 1. The coefficients of variation (CV) for pop2 across three environments were 0.34, 0.39, and 0.31, respectively. For pop1, the CV values across three environments were 0.45, 0.46, and 0.48. The absolute values of skewness and kurtosis for both pop1 and pop2 populations were less than 1, indicating a minor degree of bias. The frequency distribution of phenotypes for pop2 and pop1 closely resembled a normal distribution. Under the three environmental conditions, the broad-sense heritability of the disease severity index for BLSB was high in pop2 and pop1 at 0.93 and 0.92, respectively. Both the genotype × environment interaction variances were statistically significant. (*p* ≤ 0.01) (Table 1) The high heritability of disease severity levels and genotype × environment interaction variances were significant underscores of the reliability of identifying BLSB-resistant genes through GWAS.

**Figure 1:**
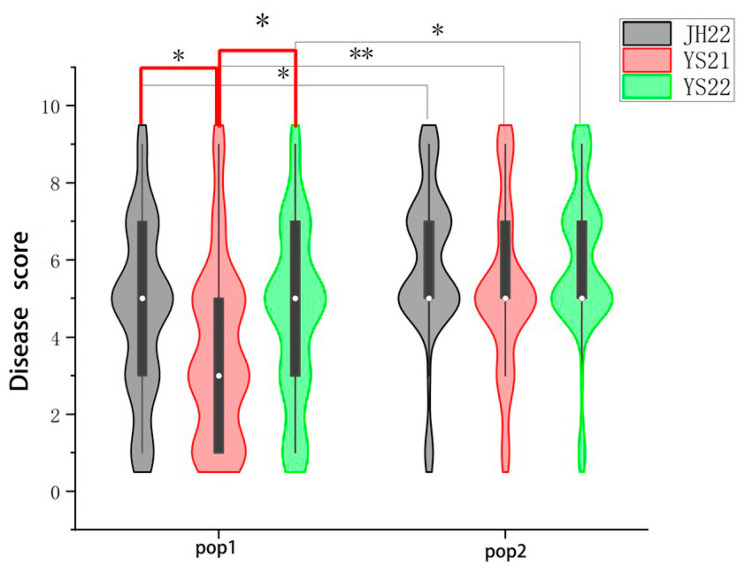
The violin plot of phenotypic distribution of RILs for pop1 and pop2 to BLSB response in three different environments (JH22, YS21, YS22). The asterisk above indicates the result of Student’s *t* test, * represents *p* < 0.05, ** represents *p* < 0.01.

As depicted in Figure 1 and Table 2, the disease scores of the majority of the RILs were more concentrated at the lower end in pop1 group than the pop2 group, indicating lower disease severity levels in pop1 than in pop2. This suggests that pop1 exhibited greater resistance to BLSB than pop2. Regarding environmental variations, significant differences in disease resistance between pop1 were observed at the YS21 environment compared to JH22 and YS22 environments, with pop2 showing no significant differences across the three environments. Notably, between pop1 and pop2 exhibited significant differences in disease resistance at the three environments JH22, YS21, and YS22.

**Figure 2:**
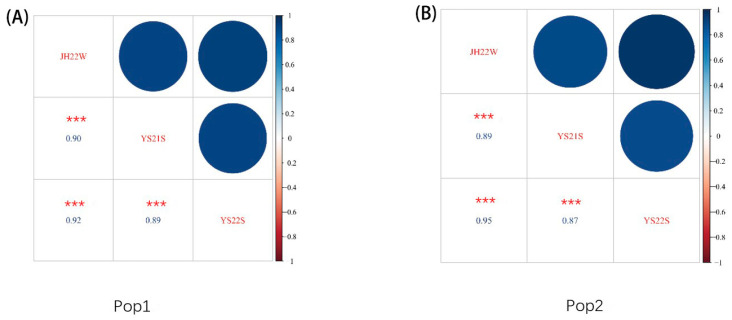
Correlation of pop1 and pop2 to BLSB response across three environments. (**A**) Correlation of pop1 to BLSB response between the JH22, YS21, and YS22 environments; (**B**) correlation of pop2 to BLSB response between the JH22, YS21, and YS22 environments. *** indicates *p* < 0.001.

The correlation analysis for BLSB across different environments within the same population is depicted in Figure 2. The correlation coefficients for disease severity levels in pop1 between the JH22, YS21, and YS22 environments were 0.90, 0.92, and 0.89, respectively. In pop2, the correlation coefficients for disease severity across the three environments (JH22, YS21, and YS22) were 0.89, 0.95, and 0.87, respectively. The consistently high correlation coefficients among the three environments indicate a significant and stable response of the RILs of pop1 and pop2 to BLSB across different environments, ensuring the reliability of the phenotyping for subsequent GWAS analyses.

### 2.2. SNP Characterization, LD Decay Distance, and Population Structure

**Figure 3:**
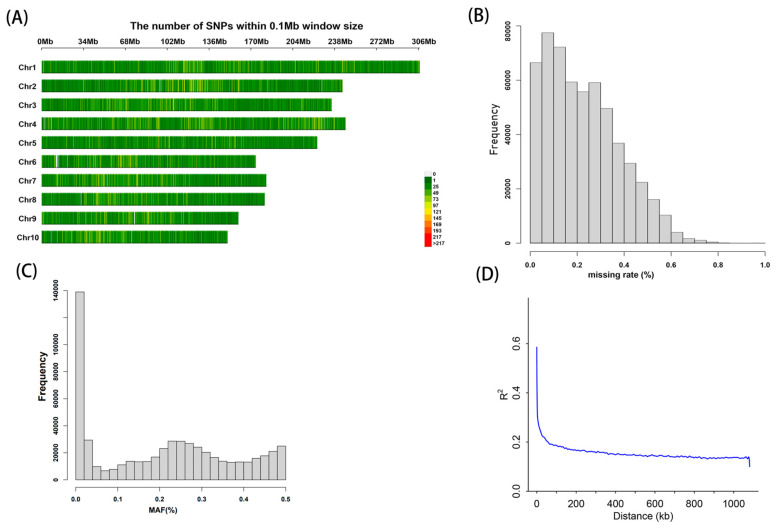
Attenuation map of genotype diversity and LD. (**A**) Chromosome-specific SNP density within 1-Mb intervals. The range of number of SNPs is indicated by a green to red scale. (**B**) Frequency distribution of missing data points of SNPs. (**C**) Distribution of minor allele frequency (MAF) of the RILs. (**D**) Genome-wide LD decay (r2) over physical distance (Kb) across all the chromosomes in 442 maize RILs.

Through Genotyping-by-Sequencing (GBS), a total of 562,212 high-quality genome-wide SNPs were identified, distributed across all ten chromosomes of maize. The heatmap in Figure 3A illustrates the marker density of SNPs across the maize chromosomes. The number of SNPs identified on chromosome 1 to 10 were as follows: 78,609, 64,672, 63,621, 72,674, 54,053, 46,111, 50,815, 47,367, 42,777, and 41,513. The highest number of SNPs was located on chromosome 1, while chromosome 9 had the fewest. The density of SNPs per mega base (Mb) for chromosomes 1 to 10 were 259.44, 265.05, 270.73, 294.23, 242.39, 265.01, 279.20, 261.70, 269.04, and 276.75, respectively. SNPs were evenly distributed across the chromosomes. In the filtered SNP dataset, the average missing rate was 0.12, and the average minor allele frequency (MAF) was 0.16, indicating the suitability of this dataset for subsequent GWAS (Figure 3B,C). We employed 562,212 SNPs to assess the linkage disequilibrium (LD) decay in the association mapping population. LD in the association mapping panel was estimated using the r2 of pairwise combinations of SNPs across the chromosomes, with the minimum threshold values set at 0.2. The estimated range of physical distance at which LD decayed was approximately 40 kb (Figure 3D). In comparison with other studies, the LD decay distance of the present study exceeded that reported in the literature. In tropical maize, the average LD decay distance across all ten chromosomes is documented as 8.14 kb [28]. For subtropical maize, at r2 = 0.2, the mean LD decay distance between chromosomes is approximately 5 to 10 kb [29]. In the research conducted by Wu et al. [30]., it was observed that the linkage disequilibrium (LD) value in temperate maize was determined to be 391 kb. Rapid LD decay indicates greater genetic diversity, fostering a more favorable genetic relationship among populations. Given that our experiment involves a population comprising both tropical and temperate maize inbreds, the LD decay rate is slow compared to tropical maize.

**Figure 4:**
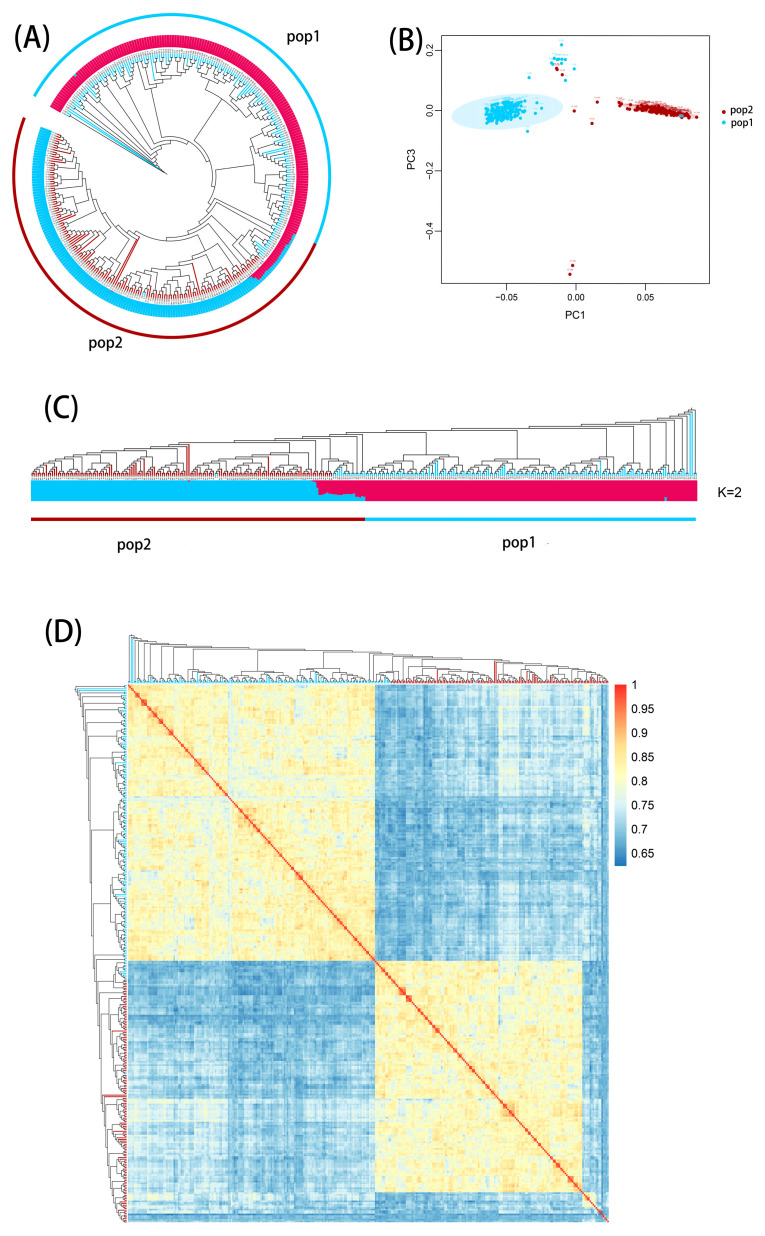
Genetic diversity analysis. (**A**) Phylogenetic tree of 442 RILs. (**B**) Principal component analysis. (**C**) Bayesian clustering plots of 442 maize inbred lines at K = 2. (**D**) Pairwise distribution of 442 maize RILs.

The population structure analysis results are depicted in Figure 4. Overall, the results of population structure, principal component analysis (PCA), and genetic distance or relatedness (Figure 4A–C) are consistent. In the PCA plot, scattered points may arise from within-population heterogeneity or outliers. Based on lineage or genetic background, the RILs can be divided into two major clusters. At K = 2 (Figure 4C), the population structure of the RILs becomes clear, with 221 RILs in pop1 and 221 in pop2. The phylogenetic tree analysis also revealed two genetic clusters, aligning with the relatedness-based population structure. The pairwise relatedness distribution chart for the 442 RILs (Figure 4D) further separates them into two distinct groups.

### 2.3. Genome-Wide Association Analysis for BLSB Resistance

The F-test revealed a significant difference between the RIL subpopulations for BLSB resistance in the two environments, Jinghong and Yanshan, and the data tend to follow a normal distribution (Figure 1). Based on the phenotypic data for BLSB disease severity of 442 RILs in the multi-parent population, and the 562,212 high-quality SNPs, GWAS analysis was conducted to identify SNPs associated with BLSB resistance. The association analysis utilized the Mixed Linear Model (MLM), which considers both the relatedness (K) and population structure as covariates to control false positives arising due to the effect of population stratification. The MLM analysis was conducted with significance levels set at −log10(*p*) > 4.5. As population structure can influence association analysis, a quantile–quantile (Q–Q) plot was employed to assess how well the adopted model accounted for population structure. The Q–Q plot indicated that this model effectively controls the population structure, with the negative log of association probability values plotted against the observed negative log of association probability values under the null hypothesis of no association. While the MLM helps reduce false-positives, it may lead to the identification of some false-negative markers. This was evident in the present study, as several significant SNPs were below the expected values in the Q–Q plot, indicating the presence of false-negatives. BLUP estimation was performed using the Lme package in RStudio (R-4.3.1) BLUP integrated disease severity levels across environments and utilized 562,212 high-quality SNPs, principal component analysis, and the Kinship matrix for GWAS analysis.

**Figure 5:**
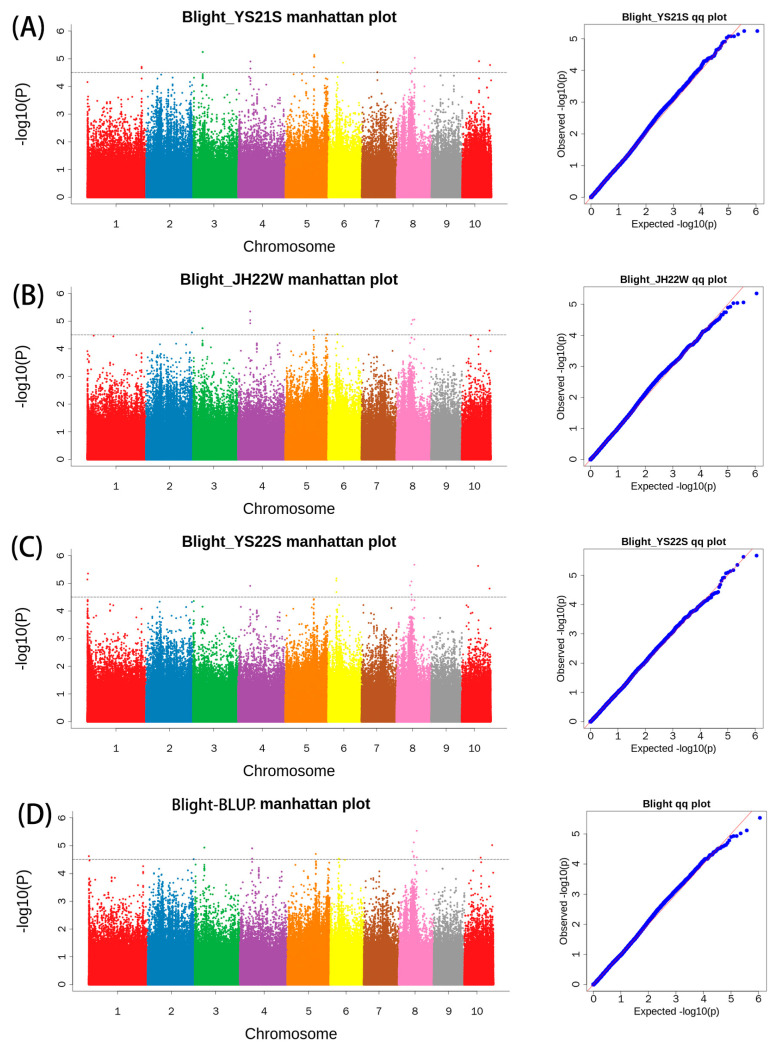
Manhattan map (**left**) and Q–Q plots (**right**) of YS21 (**A**), JH22 (**B**), YS22 (**C**), BLUP (**D**) show SNPs associated with BLSB resistance. Each dot on the left figures represents an SNP, and the black line represents the threshold of <1 × 10^−4^. Different colors represent different chromosomes The red lines on the right figures are the trend lines to which the ideal Q–Q plot in each case should correspond.

In the JH22W environment, a total of 13 SNPs significantly associated with BLSB resistance (with a significant threshold of −log10(*p*) = 4.5) were identified. These SNPs were distributed across chromosomes 2, 3, 4, 5, 6, 8, and 10 (Figure 5A). The significant SNPs explained a phenotypic variance (PVE) ranging from 3.18% to 11.71%, with an average of 5.02%. Among all the significant SNPs, chromosome 5 had the highest number (five SNPs), followed by chromosome 8 (four SNPs), while chromosomes 10 and 4 each had three SNPs, chromosome 3 had two SNPs, and the remaining chromosomes had only one.

In the YS21S environment, a total of 18 SNPs significantly associated with resistance to BLSB (with a significant threshold of −log10(*p*) = 4.5) were identified. These SNPs were distributed across chromosomes 1, 3, 4, 5, 6, 7, 8, and 10 (Figure 5B). The significant SNPs explained a phenotypic variance (PVE) ranging from 2.74% to 11.71%, with an average of 6.69%. Among all significant SNPs, chromosome 5 had the highest number (eight SNPs), while chromosomes 1, 6, and 7 had the fewest, each with one SNP.

In the YS22S environment, a total of 12 SNPs significantly associated with resistance to BLSB (with a significant threshold of −log10(*p*) = 4.5) were identified. These SNPs were distributed across chromosomes 1, 4, 6, 8, and 10 (Figure 5C). The significant SNPs explained a phenotypic variance (PVE) ranging from 4.26% to 11.71%, with an average of 8.33%. Among all significant SNPs, chromosome 8 had the highest number (five SNPs), while chromosome 4 had the fewest, with one SNP.

In the BLUP analysis, a total of 17 SNPs significantly associated with resistance to BLSB (with a significant threshold of −log10(*p*) = 4.5) were identified. These SNPs were distributed across chromosomes 1, 2, 3, 4, 5, 6, 8, and 10 (Figure 5D). The significant SNPs explained a phenotypic variance (PVE) ranging from 2.48% to 11.71%, with an average of 6.67%. Among all significant SNPs, chromosome 8 had the highest number (ten SNPs), while chromosomes 1 and 2 had the fewest, each with one SNP.

In summary, a total of 19 SNPs significantly associated with BLSB were identified across the four environments, using a significant threshold of −log10(*p*) = 4.5) (Table 3). These SNPs serve as valuable candidates for further gene identification.

### 2.4. Candidate Genes Revealed by GWAS

**Figure 6:**
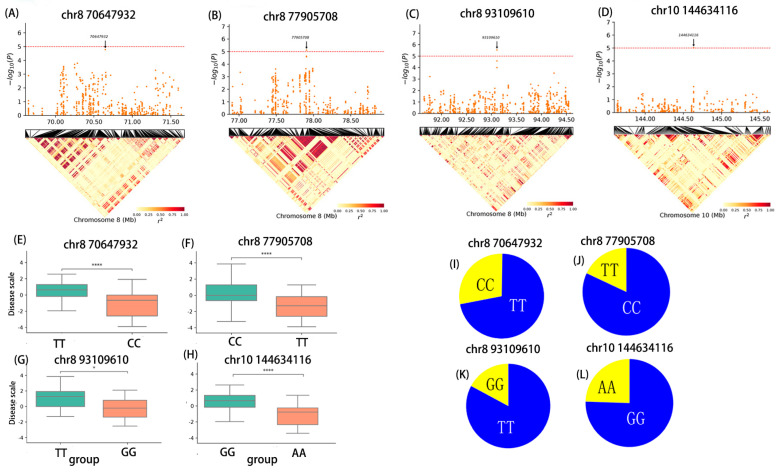
Identification of four SNPs associated with BLSB resistance, including chr8:70647932, chr8: 77905708, chr8:93109610, and chr10:144634116. Manhattan plot and LD heatmaps of chr8:70647932 (**A**), chr8:77905708 (**B**), chr8:93109610 (**C**), chr10:144634116 (**D**). (**E**–**H**), respectively, Illustrate the difference in the corresponding phenotype between the two groups of RILs. The TT haplotype of the SNP chr8:70647932 is substituted to CC, the CC haplotype of the SNP-chr8: 77905708 is substituted to TT, the TT haplotype of the SNP-chr8:93109610 is substituted to GG, the GG haplotype of the SNP-chr10:144634116 is substituted to AA. Haplotype distribution of SNP-chr8:70647932 (**I**), SNP-chr8:77905708 (**J**), SNP-chr8:93109610 (**K**), SNP-chr10: 144634116 (**L**) in the multiparent population. * represents significant *p* < 0.05, **** represents highly significant *p* < 0.0001.

Using the B73 RefGen_v4 reference genome, 23 candidate genes were identified within a 40 Kb region upstream and downstream of the 19 SNPs significantly associated with BLSB (Table 4). Table 3 provides functional annotations for these candidate genes sourced from maizeGDB. These genes are associated with maize cell membrane proteins, cell immune proteins, and cell metabolism, transport, transcriptional regulation, and structural proteins. Based on functional annotations and genes that were consistently identified across different environments (Table 3), five candidate genes with the potential to have a role in regulating BLSB resistance were identified in this study. Three of these candidate genes, namely *Zm00001d009723* (chr8:77905708) (Figure 6B, Table 4), *Zm00001d009975* (chr8:93109610) (Figure 6C, Table 4), and *Zm00001d026376* (chr10:144634116) (Figure 6D, Table 4), may play a direct or indirect role in influencing BLSB resistance in maize, with phenotypic variance (PVE) of 7.54%, 11.71%, and 4.14%, respectively. These three genes were consistently identified across four distinct environments (Table 3). The first two candidate genes are located on chromosome 8, while the subsequent one is located on chromosome 10. The functions of these three genes are primarily related to protein binding, defense against various stress stimuli, resistance to fungi, and transmembrane protein transport. Additionally, two candidate genes (*Zm00001d009566* and *Zm00001d009567*) placed in close proximity to the significant SNP positioned at chr8:70647932 were consistently identified in two environments (Figure 6A, Table 4). This SNP explained a phenotypic variance of 5.31% and both genes, located on chromosome 8, are associated with cell immune defense.

**Figure 7:**
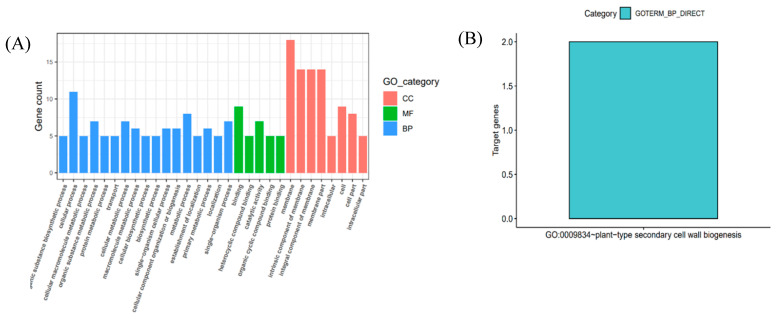
(**A**) GO annotation of candidate genes. (**B**) GO enrichment analysis of gene enrichment map with *p* < 0.05.

The 23 genes listed in Table 4 were subjected to Gene Ontology (GO) enrichment analysis (Figure 7A). These genes were associated with a total of 30 GO terms, with protein binding and catalytic activity being the most annotated GO terms in the Molecular Function (MF) category. In the Cellular Component (CC) category, the GO terms included cell membrane, intracellular membrane, cell, and intracellular, with intracellular being the most annotated term. In the Biological Process (BP) category, GO terms such as biosynthetic process, cellular process, macromolecule, organic substance, and protein metabolic process were encompassed, with cellular process emerging as the most frequently annotated term. Notably, within this category, the term GO:0009834~plant-type secondary cell wall biogenesis exhibited enrichment for two genes (Figure 7B), associated with the process of secondary cell wall formation in plants. These two genes, *Zm00001d009566* and *Zm00001d0095670*, align with previously identified candidates found in only two environments.

The haplotypes of the aforementioned SNPs are illustrated in Figure 6. For instance, the SNP at chr8:77905708 is in the intron of the candidate gene *Zm00001d009723*, with a C–T substitution (Figure 6F,J). The SNP at chr8:93109610 is positioned at 18,578 bp downstream of the candidate gene *Zm00001d009975*, with a T-G substitution (Figure 6G,K). The SNP at chr10:144634116 is located in the intron of the candidate gene *Zm00001d026376*, with G–A substitution (Figure 6H,L). The SNP at chr8:70647932 is situated 13,849 bp downstream of the candidate genes *Zm00001d009566* and *Zm00001d009567*, with a T-G substitution (Figure 6E,I). The base changes, including (C/T), (T/G), (G/A), and (T/C), at the SNP sites lead to significant differences in base compositions among RILs. These indicated that the TT, GG, AA, and CC haplotypes of the SNPs positioned at chr8:77905708, chr8:93109610, chr10:144634116, and at chr8:70647932, respectively, are favorable for reducing BLSB.

## 3. Discussion

### 3.1. The Feasibility of GWAS in this Experiment

Genetic studies of complex traits in plants have historically been challenging due to the influence of multiple minor-effect genes, often modulated by diverse genetic backgrounds or environmental conditions. Against this backdrop, genome-wide association study (GWAS) has emerged as a potent tool for dissecting the genetic factors underlying complex quantitative traits. The identification of genes resistant to bacterial leaf and sheath blight of maize (BLSB) proves to be a pertinent focus for GWAS, given its economic impact as a complex maize disease [31]. In this study, we employed 562,212 SNPs to identify 19 potential loci controlling resistance to BLSB in the multiparent population. Over the past years, GWAS has delivered promising results by uncovering novel disease-resistant loci [32,33,34]. In contrast to traditional bi-parental mapping, this approach is not influenced by the quantity of recombination events and is not constrained by the reduction in the number of allelic variants present in the parents. This method can effectively capture allelic variants underrepresented in the population [35] and accelerate the selection of allelic combinations suitable for the target environment in the donor pool [36]. However, factors such as phenotypic variation, population size, and population substructure can also influence the precision of GWAS [37]. Our results indicate that resistance to BLSB in this population is controlled by polygenes with small to moderate phenotypic effects. Therefore, the authors assert that the GWAS method is highly applicable for identifying molecular loci related to resistance against BLSB in maize and exploring candidate genes.

### 3.2. Comparison of the Results of the Present Study with the Previous Results

Previous studies have identified several candidate genes associated with resistance to BLSB. Li et al. [18] conducted GWAS and discovered a significant association between *ZmFBL41*, (on chromosome 4), encoding an F-box protein, and resistance to BLSB in maize. Using a natural population of 318 different inbred lines, the authors identified 28 SNPs located on chromosomes 1, 4, 7, and 8. The mechanism involves the interaction of *ZmFBL41* with *ZmCAD*, leading to the degradation of *ZmCAD* and disruption of lignin synthesis. This degradation weakens the plant cell wall, making it more susceptible to fungal colonization, highlighting the crucial role of the cell wall and lignin in plant immunity. The candidate genes identified in this study, *Zm00001d009566* and *Zm00001d009567*, are associated with secondary wall formation in plants and are linked to cell immune defense, indicating the credibility of these two genes.

Cao et al. [1] employed RNA sequencing to analyze the gene expression profiles of maize infected with different virulent strains of BLSB. They identified numerous differentially expressed genes, including those related to defense. Gene ontology (GO) terms related to defense were enriched in both low virulence (LVS) and high virulence (HVS) regulatory genes, such as “response to stimulus”, “defense response”, “cell wall organization”, “response to stress”, “response to biotic stimulus”, and “response to hormones”. The gene *Zm00001d009975* identified in this study exhibits similar responses, participating in defense against various stress stimuli and playing a role in resistance to fungal diseases. Additionally, a set of core genes was discovered, among which the overexpressed genes *zmac41* and *ZmBAK1* enhance maize resistance to BLSB. Chen et al. [38], through meta-analysis, identified 15 “consistency” QTLs controlling resistance to BLSB in maize, distributed on chromosomes 2, 4, 6, 8, 9, and 10. The authors also identified five hotspots (Bin2.02, 2.06, 6.02, 9.0, 9.04) for BLSB resistance. Yang [39] constructed a genetic linkage map using 125 SSR markers in a backcross (BC1) population (CML270 × 478) × CML270) developed by crossing the resistant maize inbred line CML270 with the susceptible inbred line 478. Through composite interval mapping, they detected three major QTLs for BLSB resistance, two on chromosome 1 and one on chromosome 7, explaining 18% and 20% of the phenotypic variation, respectively. Lin et al. [40] identified 11 BLSB resistance-related QTLs based on disease index, distributed on chromosomes 2, 4, 5, 8, and 9, with additive effects that explained phenotypic variance ranging from 0.0165 to 0.0545 and 20.81% to 7.29%, respectively. Chen et al. [41] analyzed QTLs and genetic effects using composite interval mapping, identifying four BLSB-resistant QTLs on chromosomes 6, 7, and 10. Two QTLs on chromosome 6 explained 12.63% and 0.27% of the phenotypic variance, while one QTL each on chromosomes 7 and 10 explained 15.21% and 5.42%, respectively. Adhikari et al. [42] conducted a population analysis using 76 polymorphic microsatellite markers. Single-marker analysis (SMA) identified markers linked to the BLSB-resistant QTL using linear regression and maximum likelihood analysis. Analysis of variance (ANOVA) revealed one major QTL on chromosome 5 and four minor QTLs on chromosomes 1, 3, 4, and 8. Liu [43], using disease index as the resistance indicator, employed composite interval mapping (LOD > 2) to locate 11 QTLs related to BLSB resistance, distributed on chromosomes 2, 4, 5, 8, and 9. The additive effects ranged from 0.0165 to 0.0545, and the phenotypic variance explained varied between 2.81% and 7.29%.

In this study, the identified genes are located on chromosomes 8 and 10, explaining a phenotypic variance ranging from 5.31% to 11.71%. While these chromosomal locations align with previous research, there are disparities in the interval. Possible explanations include: (1) the use of diverse germplasms or varieties in different studies, leading to significant variations in response to BLSB infection. This study used tropical germplasms, potentially enhancing genetic diversity; (2) substantial environmental impact on BLSB, as observed in the locations of Yanshan County and Jinghong City in Yunnan, classified as high-temperature- and high-humidity-prone areas; (3) variations in sample size and statistical methods across studies may affect GWAS accuracy. This study involved a total of 442 RILs, exceeding the sample sizes used in previous studies [44,45,46,47,48]. These considerations underscore the importance of accounting for genetic diversity, environmental factors, and methodological variations when interpreting GWAS results, highlighting the robustness of this study’s approach with its diverse tropical germplasm and larger sample size.

### 3.3. Functional Annotation of Genes Identified through GWAS

In this study, we compared the positions of SNPs significantly associated with BLSB resistance, consistently identified across four environments, with previous studies. However, no overlapping regions were found. This indicates that the BLSB resistance genes, namely *Zm00001d009723, Zm00001d009975, Zm00001d026377*, *Zm00001d009566*, and *Zm00001d009567*, identified in this study could be novel candidate genes related to BLSB resistance. These candidate genes were functionally annotated to decipher their potential role in disease resistance.

*Zm00001d009723* encodes the SET domain, characterized by a multi-helix structure with irregularly arranged long α-helices and short α-helices. This domain is widely present in various organisms, including plants, yeast, mammals, bacteria, and viruses [49] In addition to its crucial role in chromatin structure and gene activity regulation, the SET domain may also be associated with plant disease defense as it participates in regulating gene expression and chromatin modification [50]

*Zm00001d009975* encodes the PADRE domain, which is associated with plant disease defense, particularly resistant to fungi. The PADRE contains a conserved sequence motif at the N-terminus and an intrinsically disordered region with multiple phosphorylation sites at the C-terminus. Typically found in small single-domain proteins with a conserved sequence motif and an intrinsically disordered region with multiple phosphorylation sites, this domain may play a role in defense against various stress stimuli, maintaining plant health and disease resistance [51].

*Zm00001d026376* encodes the WD40 repeat, a short motif of 40 amino acids or fewer, usually terminating with a Trp-Asp (W-D) dipeptide. This motif typically comprises 7–8 blade-like β-propeller folds but may contain 4 to 16 repeat units [52]. WD-repeat proteins are widely distributed in various organisms and participate in diverse functions such as signal transduction, transcription regulation, cell cycle control, and apoptosis. They may act as platforms for protein complex assembly or mediators for protein–protein interactions, potentially involved in plant disease defense, such as the assembly of complexes like G proteins, TAFII transcription factors, and E3 ubiquitin ligases [53,54]. Some proteins containing WD40 repeat sequences may participate in plant processes related to diseases, as observed in Arabidopsis, where proteins with WD40 repeat sequences play a crucial regulatory role, including resistance and defense responses against pathogens. These proteins may play essential roles in signaling pathways, initiating the plant’s immune response against diseases.

*Zm00001d009566* and *Zm00001d009567* both encode the FAS1 domain, an extracellular module typically containing around 140 amino acid residues, found in plants, animals, and bacteria. This domain features a novel fold composed of seven-chain β-wedges and at least five α-helices, along with conserved elements related to asparagine [55,56,57]. Proteins known to contain the FAS1 domain include the human TGF-β-induced Ig-H3 protein, volvox’s major cell adhesion protein, Arabidopsis’ fasiclin-like arabinogalactan protein, mammalian stabilin protein, human extracellular matrix protein periostin, and bacterial immunogenic protein MPT70, among others. Although there is no detailed description of the disease defense-related aspects of the FAS1 domain, it can be speculated that this domain may play a role in cell adhesion and interactions, possibly related to the plant’s defense mechanisms. Cell adhesion and interactions in plants play a crucial role in disease defense as they can impact pathogen invasion and the plant’s immune response [58,59,60,61,62].

The presence of these genes and domains suggests their potential roles in plant disease defense and resistance mechanisms, involving critical biological processes such as gene regulation, protein interactions, immune responses, and cell adhesion. Further investigation into the functions of these genes and domains will contribute to a better understanding of how plants respond to various pathogens and stresses, ultimately enhancing their disease resistance capabilities.

## 4. Materials and Methods

### 4.1. Plant Materials and Population Construction

This study, conducted by our group in 2012, employed the tropical maize inbred lines CML444 and NK40-1, known for their resistance to banded leaf and sheath Blight (BLSB), as female parents. These were crossed with the temperate maize inbred line Ye107, which is susceptible to BLSB. The pedigrees, heterotic groups, and ecotypes of the three parents are detailed in Table 5. The disease scale was derived from field surveys, and the survey methodology followed the guidelines outlined in Table 6. Through a single-seed descent method over eight generations, two recombinant inbred line (RIL) subpopulations were generated: pop1 (CML444 × Ye107) and pop2 (NK40-1 × Ye107), each consisting of 221 RILs. All 442 RILs from both subpopulations were planted in Yanshan County, Yunnan Province (23°19′–23°59′ N, 103°35′–104°45′ E) in 2021 and in Yanshan County and Jinghong City (21°27′–22°36′ N, 100°25′–101°31′ E) in 2022. The experimental design followed a Randomized Complete Block Design (RCBD) with three replications per group. Each row consisted of 14 plants, with a row length of 4 m, row spacing of 0.70 m, and inter-plant spacing of 25 cm. Standard agronomical practices were followed to manage the maize experimental sites [63].

### 4.2. Disease Scoring and Calculation of Disease Index

BLSB screening of RILs of the multiparent populations was conducted in field conditions at two sites, Yanshan County (YS) and Jinghong city (JH), Yunnan Province, China, during the summer of 2021 and 2022 and the winter of 2022. The resistance to BLSB was assessed starting in the 4th week after maize dispersal. The BLSB resistance score was determined for each RIL population based on the percentage of total leaf area infected by BLSB. The screening was performed to assess BLSB resistance levels when the temperature ranged between 20 and 25 °C, and high relative humidity (over 81%) prevailed in the summer in the YS region and in the winter in the JH region of Yunnan province. These conditions created a favorable environment for the growth and spread of *Rhizoctonia solani* [7,8].

The disease severity classification for BLSB followed symptom identification criteria outlined in the ‘Manual of Corn Diseases and Insect Pests’ by Wang Xiaoming and Dai Fachao [64]. Severity assessment involved determining the largest leaf sheath lesion, with a disease score assigned to each plant on a scale of 0 to 9, as detailed in Table 6.

### 4.3. Phenotyping and Statistical Analysis

The phenotypic data were subjected to descriptive statistical analyses using SPSS (SPSS Statistics 26) and ORGIN (Orgin 2022) software. Measures such as mean, minimum, maximum, standard deviation (SD), coefficient of variation (CV), skewness, and kurtosis were calculated. Phenotypic data frequency distribution was conducted using SPSS software. Kurtosis and skewness were employed to assess the normality of the frequency distribution. Pearson’s correlation analysis was performed using the Corrplot function in R. Broad-sense heritability was calculated following the approach outlined by Knapp et al. [65,66].

$$H2=\frac{\sigma_{g}^{2}\;}{\sigma_{g}^{2}+\sigma_{ge}^{2}/e+\sigma_{\varepsilon }^{2}/re}\times 100\%$$

where $\sigma_{g}^{2}$ refers to the genetic variance, $\sigma_{ge}^{2}$ refers to the variance of genotype-environment interaction, $\sigma_{\varepsilon }^{2}$ refers to residuals, e refers to number of environments (the sum across years and locations), and r refers to the number of replications [65].

Additionally, we employed the lme4 version 1.1–30 [67] R package for calculating BLUP. The calculation formula used was

Multi-locations:

$$Y_{\mathrm{ijlk}}=\mu +{Line}_{i}+{Loc}_{j}+{\left(Line\times Loc\right)}_{\mathrm{ij}}+Rep\;{\left(Loc\right)}_{\mathrm{jl}}+\varepsilon_{\mathrm{ijlk}}$$

One-location:

$$Y_{\mathrm{ilk}}=\mu +{Line}_{i}+\varepsilon_{\mathrm{ilk}}$$

where *Y_ijlk_*, *μ*, *Line_i_*, and *Loc_l_* represent the BLSB phenotype values of each plant, intercept, *i*th line effects, and *l*th location effects, respectively. *Rep_j_* represents the *j*th replication effect, and ε*_ijlk_* represents the random effects. (*Line* × *Loc*)*_ij_* is used to display the interaction of the ith line at the *j*th location, and Rep (*Loc*)*jl* shows the nested effect of the *j*th replication within the lth location. The *Y_ilk_* is the BLSB phenotype value of each ear at one location, and ε*_ilk_* represents the random effects.

The calculated BLUP values, along with the average phenotypic data from YS and JH, were used for the subsequent GWAS.

### 4.4. DNA Extraction and Genotyping-by-Sequencing (GBS)

The genomic DNA was extracted from seedling leaves of each F8RIusing the cetyl trimethyl ammonium bromide (CTAB) method [68]. Subsequently, the isolated genomic DNA from each F8RIL was digested with the restriction endonucleases PstI and MspI (New England BioLabs, Ipswich, MA, USA), followed by ligation with barcode adapters using the T4 ligase (New England BioLabs). GBS DNA libraries were constructed and sequenced following the GBS protocol [69].

All ligated samples were pooled and purified using the QIAquick PCR Purification Kit (QIAGEN, Valencia, CA, USA). Polymerase chain reaction (PCR) amplification was carried out using primers complementary to both adaptors. Finally, the PCR products were purified and quantified using the Qubit dsDNA HS Assay Kit (Life Technologies, Grand Island, NY, USA). After selecting PCR products of 200–300 bp size using an Egel system (Life Technologies), the library concentration was estimated with a Qubit 2.0 fluorometer and the Qubit dsDNA HS Assay Kit (Life Technologies). Sequencing was performed on an Ion Proton sequencer (Life Technologies, software version 5.10.1) with P1v3 chips. The final reads were generated using TASSEL v5.0 (https://github.com/Euphrasiologist/GBS_V2_Tassel5, (accessed on 6 March 2022)) [70]. Before TASSEL analysis, 80 poly (A) bases were appended to the 3′ ends of all sequencing reads. SNPs were generated using the Genome Analysis Toolkit software (GATK-3.8) [71] by aligning with the maize B73 reference genome (B73_V4, ftp://ftp.ensemblgenomes.org/pub/plants/release-37/fasta/zea_mays/DNA, (accessed on 6 March 2022)) [72]. Following the SNP calling in the RILs, the quality of each SNP was assessed based on criteria such as minor allele frequency (MAF), the percentage of missing data points, and linkage disequilibrium. Plink v 1.9 [73] was utilized to filter the SNPs, with the parameters set to −geno 0.2 and −maf 0.05, to exclude loci with deletion rates above 10% and loci with minimum allele frequencies below 5%. In total, 562,212 high-quality SNPs were generated and annotated using the ANNOVAR software tool (v2013-05-20) [74].

### 4.5. Linkage Disequilibrium (LD), Population Structure, and LD Block Analysis

During population structure analysis, we employed a model-based clustering algorithm in ADMIXTURE Version 1.3 [75]. Preliminary analysis involved multiple runs with consecutive K values ranging from 1 to 12, with a five-fold cross-validation procedure for each K value. The most likely K value was determined using the cross-validation value of ADMIXTURE. Inbred lines with a membership probability greater than 0.5 were assigned to corresponding clusters and visualized using TBtools software v1.098727 [76]. Principal component analyses (PCA) and cluster analyses were performed in R. Linkage Disequilibrium (LD) was evaluated using Pop LD decay 3.40 software (https://github.com/BGI-shenzhen/PopLDdecay (accessed on 23 June 2023)) and Perl scripts [77]. LD decay was investigated by plotting pairwise r2 values against physical distance in base pairs, with the critical r2 for LD decay determined by keeping the minimum threshold values at 0.2, indicating the maximum significant physical distance at which LD occurs.

### 4.6. Genome-Wide Association Study

GWAS was conducted using the efficient mixed-model association (EMME) analysis method in the GEMMA (genome-wide efficient mixed-model association) package [78]. The analysis used the following mixed-model approach:

*y* = *Xa* + *Sb* + *Km* + *e*;

where *y* represents the phenotype, and a and b are fixed effects denoting marker effects and non-marker effects, respectively. m represents unknown random effects. The incidence matrices for *a*, *b*, and *m* are denoted by *X*, *S*, and *K*, respectively, while e is a vector of random residual effects. To account for population structure, the top three principal components (PCs) were used to construct the *S* matrix, and the kinship (*K*) matrix was built using the matrix of simple matching coefficients. The genetic relationship between individuals was modeled as a random effect using the *K* matrix. A significant *p*-value threshold of *p* < 1 × 10^−6^ was set to control the type 1 error during association analysis.

We used PLINK [73] to calculate independent markers with the parameter -indep-pairwise 50 5 0.2. A significance threshold of −log10(*p*) > 4.5, calculated using the formula −log10(1/SNP numbers), was employed to identify significant SNPs associated with maize BLSB resistance. SNP loci meeting or exceeding the threshold were extracted using bedtools v1.7 [79] Based on the B73 v4 reference genome and annotation information, candidate genes associated with BLSB in maize were identified within a 40 kb upstream and downstream region of the significantly associated SNPs. The decision to use a 40 kb region for screening candidate genes was based on a plateau observed in the LD decay plot, where the r2 values plateaued at 40 kb.

### 4.7. Gene Predictive Analysis

Based on the estimation of LD decay rate, genes situated within 40 kb upstream and downstream of the significant SNPs underwent functional annotation, Subsequently, relevant gene functional annotation information was retrieved from the maizeGDB database (http://www.maizegdb.org (accessed on 8 August 2023)). The determination of physical positions for both genes and SNPs relied on the maize B73 RefGen_V4 reference genome. GO analysis for the identified candidate genes from the GWAS was executed utilizing the DAVID tool (https://www.DAVID.com/tools (accessed on 10 August 2023)) [80] and the agrigo tool (https://www.agrigo.com/tools (accessed on 10 August 2023)) [81]. Data visualization was conducted using R Studio. This annotation process aided in further validation and identification of candidate genes associated with the target traits acquired through GWAS analysis. The *p*-values for GO analyses were computed using the following formula [82].

$$P=1-\sum_{i=0}^{K-1}\frac{\left(\begin{array}{c} M\\ i \end{array}\right)\times \left(\begin{array}{c} N-M\\ n-i \end{array}\right)}{\left(\begin{array}{c} N\\ n \end{array}\right)}$$

*N*: Total number of samples;

*M*: The number of “specific categories” in the total sample;

*n*: A random number drawn from the total sample;

*k*: Take the number of samples that belong to a “specific category”.

### 4.8. Haplotype Analysis

Haploview v4.2 software was used to analyze the genes consistently detected in multiple environments or those with functions related to BLSB.

## 5. Conclusions

In this study, two F8 RIL subpopulations were developed by crossing the inbred parent Ye107 with inbred lines CML444 and NK40-1. A GWAS, utilizing 562,212 high-quality SNPs, identified nineteen SNPs significantly associated with BLSB resistance. Subsequently, 23 candidate genes related to BLSB resistance were identified within a 40 kb upstream and downstream region of these SNPs. By employing genome databases such as maizeGBD and NCBI, five novel candidate genes, including *Zm00001d009723*, *Zm00001d009975*, *Zm00001d026376*, *Zm00001d009566*, and *Zm00001d009567*, were validated to confirm their potential roles in BLSB resistance. These genes are implicated in diverse activities encompassing cellular metabolism, transport, transcriptional regulation, and structural proteins. They are involved in various functions, such as protein binding, defense against multiple stress stimuli, resistance to fungal infections, protein transmembrane transport, and enhancement of immune defense. In conclusion, the insights gained from this study could significantly contribute to a deeper comprehension of the regulatory mechanisms underlying BLSB resistance in maize. The identified SNPs and candidate genes hold promise for aiding breeders in selecting maize varieties with enhanced BLSB resistance.

## Figures and Tables

**Table 1:** Descriptive statistics of the two RIL subpopulations for reactions against BLSB.

Populations (Sample Number.)	Env.	Range	Mean	SD	CV (%)	Skewness	Kurtosis	Variations			H^2^ (%)
								G	E	G*E	
Pop2 (221)	JH 22 W	1–9	5.59	1.922	0.34	−0.518	0.713				0.93
	YS 21 S	1–9	5.25	2.073	0.39	0.163	−0.061	3.42 *	0.25 *	0.3 *	
	YS 22 S	1–9	5.98	1.865	0.31	−0.408	0.757				
Pop1 (221)	JH 22 W	1–9	4.66	2.18	0.45	−0.035	−0.586				
	YS 21 S	1–9	3.62	2.252	0.46	0.661	−0.133	4.14 *	0.49 *	0.118 *	0.92
	YS 22 S	1–9	4.58	2.226	0.48	−0.04	−0.638				

W represents winter, S represents summer. Env. represents environment. Range represents the range of disease grade of plants within the population. SD represents standard deviation. CV represents coefficient of variation. H^2^ represents heritability. * represents *p* < 0.05.

**Table 2:** Analysis of variance for pop1and pop2 evaluated. * represents *p* < 0.05.

Pop	df	Sum of Squares	F-Value	*p*	Error
Pop1	2	5.60	14.9	0.0227 *	0.52
Pop2	2	4.12	7.98	0.056	0.38

**Table 3:** List of 19 significantly associated SNPs identified across four environments.

No.	Chr	Physical Position	Threshold (−log10(*p*) = 4.5)	Allele	Environments
1	1	442415	4.62	C/T	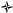 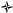
2	1	282946785	4.71	G/A	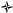
3	2	239027952	4.51	C/T	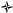 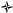
4	3	50067223	4.92	A/G	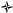
5	4	63379553	4.90	C/T	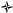
6	4	63306693	4.90	T/A	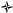
7	5	147529611	4.69	T/C	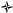 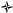
8	5	148963157	5.13	C/T	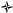
9	5	147757336	4.66	T/C	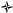
10	5	217509599	4.51	C/T	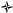
11	6	42410465	4.52	A/G	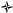
12	6	46848365	4.53	C/T	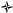 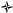
13	8	70647932	4.78	C/T	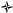 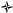
14	8	77905708	5.11	C/T	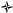 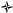 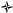 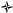
15	8	93109610	5.53	T/G	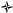 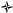 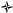 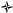
16	8	83921201	5.04	C/T	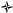
17	10	144634116	4.81	G/C	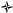 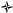 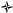 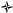
18	10	87410402	4.91	C/T	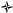
19	10	84908655	5.62	C/T	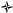

No. represents number, chr represents chromosome. Environments represents the point repeatedly located in the four environments, 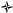

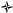

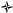

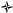
 represents the SNPs identified across the four environments repeatedly located, 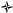

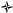
 represents the SNPs identified in both environments repeatedly located, 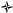 represents the SNPs identified in one environment.

**Table 4:** Candidate genes identified through screening of the significantly associated SNPs and functional annotation.

Chr	Position	Gene ID	PVE	Protein	Function
1	442415	Zm00001d027254	10.41%	Uncharacterized	unknown
1	282946785	Zm00001d034064	8.90%	PAT complex subunit Asterix	protein insertion into ER membrane
2	239027952	Zm00001d007758	4.45%	Overlapping homologous superfamilies	intracellular protein transport
3	50067223	Zm00001d040563	5.54%	Uncharacterized	
4	63379553	Zm00001d050063	2.48%	Zinc finger, RING-type	overlapping homologous superfamilies
4	63306693	Zm00001d050062	6.57%	Uncharacterized	unknown
5	147529611	Zm00001d016156	7.59%	1,3-beta-glucan synthase component FKS1-like, domain-1 InterPro entry	putative callose synthase 8
5	148963157	Zm00001d016183	5.15%	Coatomer, epsilon subunit	retrograde vesicle-mediated transport, Golgi to endoplasmic reticulum
5	147757336	Zm00001d016161	5.08%	CRIB domain	overlapping homologous superfamilies
5	217509599	Zm00001d018257	3.45%	unknown	unknown
		Zm00001d018258		SANT/Myb domain	overlapping homologous superfamilies
		Zm00001d018259		Ubiquitin-like protein Atg12	autophagosome assembly
		Zm00001d018260		Glutamine-Leucine-Glutamine, QLQ	regulation of DNA-templated transcription
6	42410465	Zm00001d035715	7.54%	Transposase, Tnp1/En/Spm-like	unknown
6	46848365	Zm00001d035769	3.40%	Chorismate mutase, AroQ class, eukaryotic type InterPro entry	aromatic amino acid family biosynthetic process
8	70647932	Zm00001d009566	5.31%	FAS1 domain	bacterial immunogenic protein MPT70 (1 FAS1 domain)
		Zm00001d009567		FAS1 domain	bacterial immunogenic protein MPT70 (1 FAS1 domain)
8	77905708	Zm00001d009723	7.54%	SET domain	protein binding
8	83921201	Zm00001d009823	5.01%	Lateral organ boundaries, LOB	unknown
8	93109610	Zm00001d009975	11.71%	PADRE domain	this domain is associated with plant defense upon diverse stress stimulus and has a role in disease resistance to fungus
10	144634116	Zm00001d026376	4.14%	WD40 repeat	protein binding
10	87410402	Zm00001d024778	6.10%	Proteolipid membrane potential modulator	transmembrane transport
10	84908655	Zm00001d024717	10.81%	unknown	unknown

Chr represents chromosome, PVE represents phenotypic variation explained.

**Table 5:** Parental lines used to develop the multiparent population.

Parent	Pedigree	Heterotic Group	Ecotype	Disease Scale
Ye107	Derived from US hybrid DeKalb XL80	Reid	Temperate	9
CML444	P43C9-1-1-1-1-1-BBBB-1-1-2-5-1(DH)	nonReid	Tropical	3
NK40-1	Derived from US hybrid	Reid	Tropical	3

DH, doubled haploid.

**Table 6:** Disease scoring for BLSB.

Scale	Reaction Category	Disease Index	Symptoms
0	Immune (IM)	0	Symptom-free throughout the entire plant
1	Highly Resistant (HR)	0.1~20.0	Disease manifestation on the fourth leaf sheath below the ear and subsequent lower leaf sheaths
3	Resistant (R)	20.1~40.0	Disease manifestation on the third leaf sheath below the ear and subsequent lower leaf sheaths
5	Moderately Resistant (MR)	40.1~60.0	Disease manifestation on the second leaf sheath below the ear and subsequent lower leaf sheaths
7	Susceptible (S)	60.1~80.0	Disease manifestation on the first leaf sheath below the ear and subsequent lower leaf sheaths
9	Highly Susceptible (HS)	80.1~100.0	Disease symptoms manifest in the leaf sheaths above the ear.

## Data Availability

The data presented in this study are available on request from the corresponding author.
